# Modulatory Impact of Lamiaceae Metabolites on Apoptosis of Human Leukemia Cells

**DOI:** 10.3389/fphar.2022.867709

**Published:** 2022-06-15

**Authors:** Izabela Berdowska, Bogdan Zieliński, Małgorzata Matusiewicz, Izabela Fecka

**Affiliations:** ^1^ Department of Medical Biochemistry, Wrocław Medical University, Wrocław, Poland; ^2^ Department of Pharmacognosy and Herbal Medicines, Wrocław Medical University, Wrocław, Poland

**Keywords:** Jurkat, apoptosis, *Thymus vulgaris L.*, *Thymus serpyllum L.*, *Origanum majorana L.*, *Mentha* × *piperita L.*, caffeic acid (CAS no: 331-39-5), rosmarinic acid (CAS no: 20283-92-5)

## Abstract

Lamiaceae species are rich sources of biologically active compounds which have been applied in medicine since ancient times. Especially their antineoplastic properties have been thoroughly studied with respect to their putative application in chemoprevention and adjuvant therapy of cancer. However, the most known biological effects of Lamiaceae have been ascribed to their essential oil fractions, whereas their (poly)phenolic metabolites being also abundant in these plants, are much less recognized, nevertheless contributing to their beneficial properties, such as anti-cancer actions. The aim of this study was to evaluate the impact of dried aqueous extracts from common thyme (*Thymus vulgaris* L.) (ExTv), wild thyme (*Thymus serpyllum* L.) (ExTs), sweet marjoram (*Origanum majorana* L.) (ExOm), and peppermint (*Mentha* × *piperita* L.) (ExMp), as well as (poly)phenolic compounds: caffeic acid (CA), rosmarinic acid (RA), lithospermic acid (LA), luteolin-7-O-β-glucuronide (Lgr), luteolin-7-O-rutinoside (Lr), eriodictyol-7-O-rutinoside (Er), and arbutin (Ab), on unstimulated Jurkat cells, in comparison with their effect on staurosporine-stimulated Jurkat cells. Jurkat T cells were incubated with different concentrations of ExTv, ExTs, ExOm, ExMp, Lgr, LA, Er, Lr, RA, CA, or Ab. Subsequently, staurosporine was added to half of the samples and flow cytometry combined with fluorescence-activated cell sorting analysis was conducted, which allowed for the selection of early and late apoptotic cells. Both ExTs and ExOm stimulated apoptosis of Jurkat cells and enhanced the proapoptotic effect of staurosporine. Conversely, ExTv and ExMp demonstrated no clear effect on apoptosis. CA and RA raised the staurosporine-induced apoptotic effect. The impact of Er and Lgr on Jurkat cells showed fluctuations depending on the compound concentration. Neither Er nor Ab altered staurosporine-induced apoptosis in Jurkat cells, whereas Lgr seemed to weaken the proapoptotic action of staurosporine. The most evident observation in this study was the pro-apoptotic action of ExTs and ExOm observed both in staurosporine-unstimulated and stimulated Jurkat cells. Additionally, an enhancement of staurosporine-induced apoptosis by caffeic and rosmarinic acids was reported. Therefore, it might be concluded that these are the mixtures of biologically active polyphenols which often exert more pronounced beneficial effects than purified molecules.

## 1 Introduction

The plant kingdom is a cornucopia of biologically active metabolites which in different forms have been applied in medicine since ancient times. Mounting evidence suggests that a high-polyphenol diet is associated with a decreased risk of a variety of disorders including obesity (Guo et al., 2017) (which leads to metabolic syndrome and atherosclerosis), diabetes (Palma-Duran et al., 2017; Pham et al., 2019), cardiovascular diseases (Yamagata, 2019), and cancers (Miyata et al., 2019; Gu et al., 2020). Therefore, especially plant polyphenols have raised attention in the scientific field engaged in the search for antioxidative and anti-inflammatory compounds which could be applied as a supplementary treatment in multiple diseases. Also, the biotechnological strategies (mainly plant cell cultures) aimed at the production of polyphenols with required features (like anti-cancer properties) are of great importance, as recently discussed by Bulgakov et al. (2018). (Poly)phenolic compounds are structurally characterized by benzene (or another aromatic ring) with one or more attached hydroxyl/phenol groups. This diverse class comprises, among others, phenolic acids (e.g., caffeic, rosmarinic, salvianolic, and lithospermic acids, also known as caffetannins), flavonoids (e.g., eriocitrin, diosmin, or hesperidin), anthocyanins, lignans, stilbenes, tannins, coumarins, and hydroquinone derivatives (e.g., arbutin). Anti-cancer functions of these plant-derived compounds have been recently addressed in some review studies (Sharma et al., 2018; Swamy et al., 2018; Hazafa et al., 2020; Lachance et al., 2020; Saeedi et al., 2021) with reference to their modulatory effects on hallmarks of cancer (the major characteristics enabling neoplastic cells to evade the host organism defense systems) (Lachance et al., 2020). A rich source of polyphenolic molecules are plants accounted to Lamiaceae (or mint family; the biggest family in the order Lamiales) encompassing over 230 genera with above 7,000 species (Zhao et al., 2021). Health-beneficial properties of Lamiaceae metabolites have been recently reviewed in some publications (Michel et al., 2020; Patrignani et al., 2021). The first team has addressed the latest data on the protective actions of essential oils and phenolic extracts against oxidative, inflammatory, neoplastic, microbial, and neurodegenerative processes and the latter has discussed the cardiovascular protective role of Lamiaceae plants extracts and biologically-active compounds. Additionally, the antifungal properties of Lamiaceae-derived essential oils have been reviewed by Karpiński et al. (2020). Generally, in comparison with essential oils, less scientific information is available on the aqueous extracts and/or isolated polyphenols originating from Lamiaceae plants.

In the present work, the pro-apoptotic potential of polyphenolic metabolites derived from four Lamiaceae species; common thyme (*Thymus vulgaris* L.; *Tv*), wild thyme (*Thymus serpyllum* L.; *Ts*), sweet marjoram (*Origanum majorana* L*.*; *Om*), and peppermint (*Mentha × piperita* L.; *Mp*), against human leukemia cells was estimated. These plants are broadly applied in many national cuisines (including Polish gastronomy), being popular spices and herbal teas, as well as are accounted as medicinal herbs (enlisted in the Community herbal monographs of the Committee on Herbal Medicinal Products in the European Medicines Agency). They have been applied in the treatment of a variety of ailments, focusing on gastrointestinal and respiratory tract disorders in European countries including Poland (European Medicines Agency 2013; European Medicines Agency 2016; European Medicines Agency 2020; Bruneton et al., 1999; McKay et al., 2006; Mahendran et al., 2020; Jarić et al., 2015. Apart from the abundance of essential oils being responsible for multiple beneficial activities of these herbs, they contain polyphenolic compounds including caffeic acid (CA), rosmarinic (RA), luteolin-7-*O*-glucuronide (Lgr), luteolin-7-*O*-rutinoside (Lr), and eriodictyol-7-*O*-rutinoside (Er) (present in all four investigated species), and arbutin (Ab) (detected in sweet marjoram and common thyme), and lithospermic acid (LA) (in wild thyme and peppermint) (Fecka and Turek 2007 and Fecka and Turek (2008). The exact chemical composition in terms of common thyme and peppermint polyphenols identified using LC-MS has been described in recent publications by Bodalska et al. (2020) and Bodalska et al. (2021). An extensive discussion on the activity and composition of sweet marjoram is presented in Bouyahya et al. (2021) and Taamalli et al. (2015).

The aim of this study was to evaluate the impact of dried aqueous extracts from common thyme (*Thymus vulgaris* L.) (ExTv), wild thyme (*Thymus serpyllum* L.) (ExTs), sweet marjoram (*Origanum majorana* L.) (ExOm), and peppermint (*Mentha × piperita* L.) (ExMp), and polyphenolic compounds: CA, RA, LA, Lgr, Lr, Er, and Ab on unstimulated Jurkat cells, in comparison with their effect on staurosporine stimulated cells, with respect to their influence on cell death *via* apoptosis/necrosis. Moreover, the observed effects were estimated in accordance with the percentage composition of (poly)phenols, and in comparison with isolated compounds.

## 2 Materials and Methods

### 2.1 Chemicals

Water was glass-distilled and deionized. Acetonitrile was LC gradient grade. The organic solvents used in the preparation of extracts and (poly)phenol solutions were of analytical grade (Chempur, Poland). Methanol, acetonitrile, 98-100% formic acid, RPMI-1640 (R8758) medium, and staurosporine were purchased from Merck–Sigma–Aldrich (Sigma–Aldrich Sp. z o.o., Poznań, Poland). Fetal bovine serum (FBS) was obtained from Panalytica/Cambrex. Annexin V-FITC Kit was obtained from BenderMedSystems. All other reagents, unless stated otherwise, were of analytical grade and purchased from Chempur (Poland).

### 2.2 Plant Material and Extract Preparation

The dried plant material: *Thymus vulgaris* L. herb (Tv), *Thymus serpyllum* L. herb (Ts)*, Origanum majorana* L. herb (Om) (syn. *Majorana hortensis* Moench.), and *Mentha × piperita* L. leaves (Mp) were purchased from the herbal company KAWON (Poland) certified GMP and ISO 9002. To obtain aqueous extracts, boiling distilled water (350 ml) was poured over the powdered herbs (5 g), stirred, and after 30 min filtered through filters (Whatman No.1, United Kingdom). The filtrates (200 ml) were acidified with formic acid (1 ml) and applied to octadecyl columns (2 × 7 cm, BAKERBOND Octadecyl 40 μm Prep LC Packing, J.T. Baker, United States), and then the polyphenols were eluted with methanol (100 ml). Methanol was distilled from the eluates at 40°C (Rotavapor R-300, BÜCHI Labortechnik AG, Switzerland). The concentrated eluates were allowed to dry, yielding the dried aqueous extracts: ExTv, ExTs, ExOm, and ExMp.

### 2.3 Standard Solutions of Polyphenols

Luteolin-7-*O*-β-glucuronide (Lgr) (syn. luteolin-7-*O*-β-glucuronoside; CAS No: 29741-10-4) and lithospermic acid (LA) (syn. lithospermic acid A; CAS No: 28831-65-4) had been isolated previously from the wild thyme herb; eriodictyol-7-*O*-rutinoside (Er) (syn. eriocitrin; CAS No: 13463-28-0), and luteolin-7-*O*-β-rutinoside (Lr) (syn. scolymoside; CAS No: 20633-84-5) from the peppermint leaf. Structures of isolated compounds had been confirmed by spectroscopic studies (^1^H NMR, ^13^C NMR, HMQC, and HRESI-MS) (Fecka and Turek 2007 and Fecka and Turek 2008).

Arbutin (Ab) (CAS No: 497-76-7) was bought in Fluka (Switzerland) and caffeic acid (CA) (CAS No: 331-39-5) in Koch-Light Laboratories (United Kingdom). Rosmarinic acid (RA) (CAS No: 20283-92-5), apigenin-7-*O*-β-glucoside (CAS No: 578-74-5), luteolin-7-*O*-β-glucoside (Lg) (CAS No: 5373-11-5), eriodictyol-7-*O*-β-glucoside (CAS No: 38965-51-4), apigenin-7-*O*-rutinoside (Agr) (syn. isorhoifolin) (CAS No: 552-57-8), diosmetin-7-*O*-rutinoside (syn. diosmin) (CAS No: 520-27-4), hesperetin-7-*O*-rutinoside (syn. hesperidin) (CAS No: 520-26-3), and naringenin-7-*O*-rutinoside (syn. narirutin) (CAS No: 14259-46-2) were from Extrasynthese (France).

Stock standard solutions of polyphenols (1 mg/ml) were prepared by dissolving an accurate amount of individual compounds in methanol and filtered through a 0.45 or 0.22 μm Millex Syringe Filter (Millipore, United States). The working standard solutions of polyphenols (0.01–0.3 mg/ml) for chromatographic investigation were obtained by dilution with 50% aq. methanol (V/V) and stored at –20°C.

### 2.4 Identification and Quantification of (Poly)phenols

(Poly)phenolic compounds were identified and quantified in dried aqueous extracts using liquid chromatography methods—UHPLC-MS and HPLC-PDA. These methods had been previously described by Fecka and Turek (2007), Fecka and Turek (2008), Berdowska et al. (2013), Bodalska et al. (2020), and Bodalska et al. (2021).

A Thermo Scientific Ultimate 3000 UHPLC instrument (Thermo Fisher Scientific, United States) was used to identify phenolic acids, flavonone, flavanone, and hydroquinone glycosides. The UHPLC-MS system consisted of quaternary pumps LPG-3400RS set with a vacuum degasser, autosampler WPS-3000TRS, column oven TCC-3000SD, and photodiode array detector DAD-3000. Detection was performed using a high-resolution mass spectrometer (ESI-qTOF Compact; Bruker Daltonics, Germany). (Poly)phenols were separated on a Kinetex RP-18 column (150 mm × 2.1 mm, 2.6 µm) (Phenomenex, United States) equipped with a dedicated precolumn. UHPLC-MS was operated in negative mode. The resulting mass spectra were analyzed using Data Analysis 4.2 software (Bruker Daltonics). Ions were collected in the scan range of 50-2200 *m/z*. Elution was performed using a gradient of acetonitrile and water with 0.1% formic acid (V/V). For other data, see Bodalska et al. (2020).

The quantification of (poly)phenols was carried out using HPLC-PDA system (Smartline, Knauer, Germany) equipped with a pump (Managare 5000), dynamic mixing chamber V7119-1, photodiode array detector PDA 2800, and manual 6-port-2-channel injection valve (A1366). Data were processed using EuroChrom^®^ for Windows Basic Edition V3.05 (V7568-5). The separation was achieved on the C18 Hypersil GOLD column (250 mm × 4.6 mm, 5 μm), with C18 Hypersil GOLD precolumn (10 mm × 4.6 mm, 5 μm) (Thermo Scientific, United Kingdom). (Poly)phenols were monitored at 254, 280, 320, and 360 nm, and UV/Vis spectra from 200 to 600 nm (scanned at a rate of 8 nm per second) were recorded for peak characterization. The binary eluent system consisted of solvent A—5% formic acid in acetonitrile (V/V) and solvent B—5% formic acid in water (V/V). Eluents were vacuum degassed with ultrasonication prior to usage. The following gradient program of eluants at a flow rate of 0.9 ml/min was used: 0–25 min, 10–40% A in B; 25–30 min, 40-70% A in B; 30–50 min, 70% A in B. Then the system returned to the initial setting and was washed with 10% A in B until the system was stabilized before the next analysis. The injection volume for all samples was 20 ml. All chromatographic experiments were performed at 22°C (column-thermostat JEATSTREM 2 PLUS ser.no. 220215; Knauer, Germany). Quantification was performed by external calibration (6 measurement points, 0.01–0.3 mg/ml). The HPLC-PDA method was completely validated as described earlier (Fecka and Turek, 2008; Berdowska et al., 2013).

The dried aqueous extracts were dissolved in 50% aq. methanol (V/V) to a concentration of 0.5–1.2 mg/ml immediately before analysis and filtered (0.45 and 0.22 μm Millex Syringe Filters). The polyphenol content (%, m/m) was expressed as the mean value from the analyses of three independent solutions of each extract, in duplicate (n = 2 × 3).

### 2.5 Cell Culture

Human Jurkat T leukemia cell line (Jurkat PCM-TC 48 stored in the Institute of Immunology and Experimental Therapy in Wrocław, Poland) was grown in cell medium fluid reaching the concentration of 10^6^ cells/ml. The cells were cultured in RPMI-1640 (R8758) medium supplemented with 10% FBS at 37°C in the atmosphere of 5% CO_2_ until a monolayer was formed (the second phase of the growth curve), avoiding the phase of cells overlapping.

### 2.6 Flow Cytometry Analysis

Jurkat T cells suspended in RPMI-1640 medium were placed in 24-well flat-bottom plates at the concentration of 4 × 10^5^ cells per well. Subsequently, they were incubated for 20 h with dried aqueous extracts obtained from Ts, Tv, Om, and Mp, or with individual compounds: flavonoid glycosides (eriocitrin = eriodictyol-7-*O*-rutinoside, scolymoside = luteolin-7-*O*-rutinoside, luteolin-7-*O*-β-glucuronide), phenolic acids (caffeic acid, rosmarinic acid, and lithospermic acid), and arbutin. Initially, the stock solutions of the examined substances were prepared by dissolving an appropriate amount of each of them in distilled water, and then required dilutions were made by mixing the stock solutions with the growth medium. In the case of herb extracts, the studied concentrations were as follows: 10, 50, and 100 μg/ml. The polyphenolic compounds were examined at the concentrations of 9, 45, and 90 μM. Subsequently, flow cytometry analysis was performed with the application of the human Annexin V-FITC Kit according to the procedure delivered by the manufacturer. The principle of this method is based on the affinity of annexin V to phosphatidylserine (PS), a membrane phospholipid which in apoptotic cells undergoes translocation from the inner to the outer leaflet of the plasma membrane. Therefore, PS exposition on the cell surface revealed by fluorescently labeled Annexin V allows for detection of the apoptotic cells subpopulation. To discriminate between early apoptotic cells, and late apoptotic/secondary necrotic cells, Jurkat T cells were concurrently stained with propidium iodide (PI) which does not penetrate in viable or early apoptotic cells but enters the cells in the phase of late apoptosis or secondary necrosis, as well as late necrotic/dead cells.

In short; Jurkat T cells were washed in PBS buffer, resuspended in the binding buffer, then Annexin V-FITC was added, and the cells were incubated for 10 min at the room temp. Finally, the cells were washed in PBS buffer, and propidium iodide (PI) was added. Subsequently, FACS analysis was performed with FACSCalibur (Becton Dickinson) using the Cell Quest program. With the application of the WinMDI program, four populations of the cells were distinguished: A-/PI- (Annexin V negative/PI negative—vital cells); A+/PI- (Annexin V positive/PI negative—cells in early stages of apoptosis); A+/PI+ (Annexin V positive/PI-positive—cells in late stages of apoptosis/secondary necrosis); A-/PI+ (Annexin V negative/PI-positive—necrotic or mechanically damaged cells).

In parallel experiments, Jurkat T cells were incubated with the abovementioned substances at the same concentrations for 17.5 h. Subsequently, staurosporine was added to all of the samples to the final concentration of 1 μg/ml, and the samples were incubated for 2.5 h. Finally, flow cytometry analysis with the human Annexin V-FITC Kit was conducted, as described above.

In all of the experiments, the control samples contained Jurkat T cells which underwent an analogical procedure with the exception that the examined substances were replaced by RPMI-1640 medium. The untreated Jurkat T cells were applied as a reference negative control (= 0 (NC)), whereas Jurkat T cells treated exclusively with staurosporine were used as a reference positive control (= S (PC).

### 2.7 Statistical Analysis

The results obtained in flow cytometry come from three independent experiments. The data were analyzed using a one-way analysis of variance (ANOVA). To address the differences between groups, the Tukey-Kramer test for multiple comparisons was performed. All effects were regarded as significant at the significance level of *p* < 0.05.

## 3 Results

### 3.1 Chemical Composition of Dried Aqueous Extracts

**FIGURE 1:**
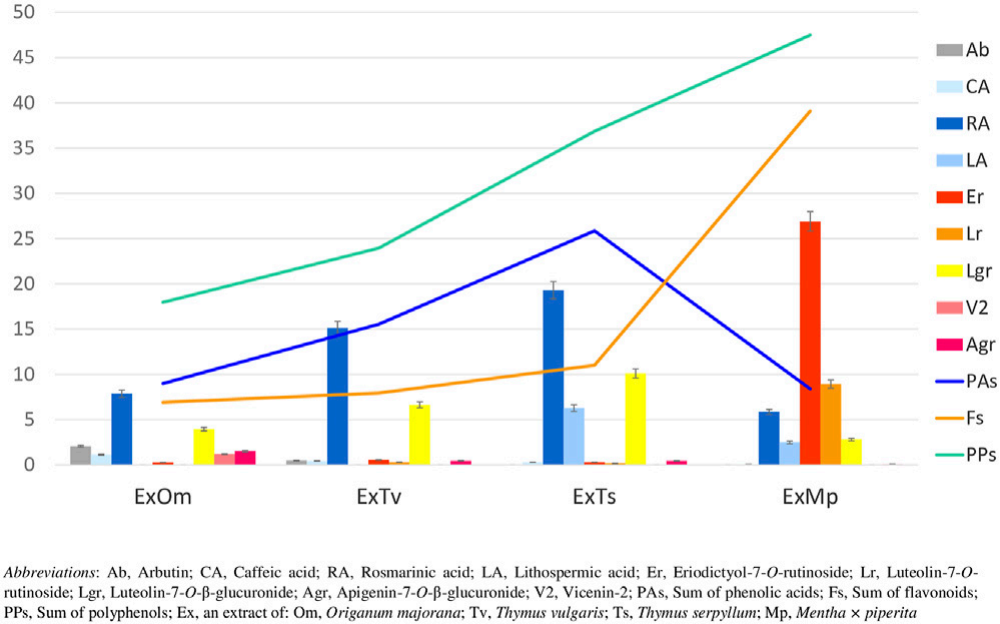
Content of polyphenols (%) in dried aqueous extracts of marjoram (ExOm), thyme (ExTv), wild thyme (ExTs), and peppermint (ExMp). The linear graphs show the sum of labeled compounds from each polyphenol group. The bar graphs represent individual polyphenol compounds.

**FIGURE 2:**
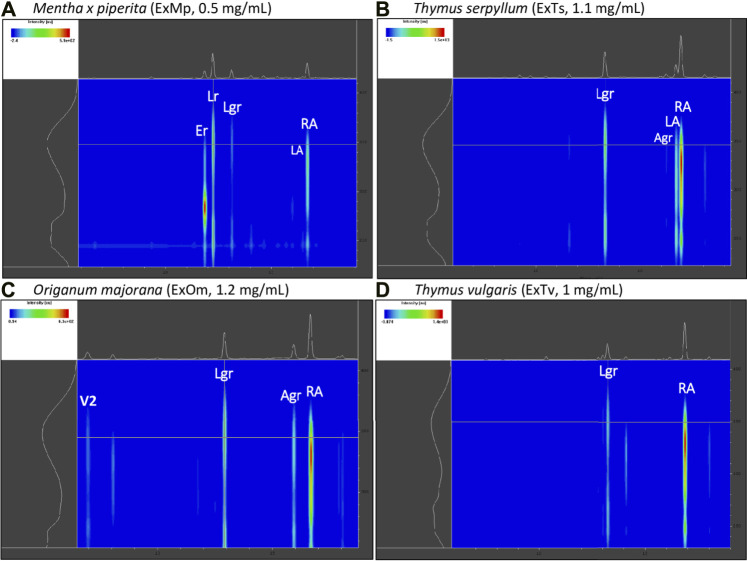
Exemplary 2D HPLC-PDA chromatograms (range: 6–20 min/230-410 nm) of the analyzed extracts with the main polyphenols highlighted. **(A)** peppermint extract (ExMp); **(B)** wild thyme extract (ExTs); **(C)** origanum majorana extract (ExOm); **(D)** common thyme extract (ExTv).

The chemical composition of dried aqueous extracts from *Thymus vulgaris* L. (ExTv), *Thymus serpyllum* L. (ExTs), *Origanum majorana* L. (ExOm), and *Mentha × piperita* L. (ExMp) was analyzed by UHPLC-MS and HPLC-PDA. The main (poly)phenolic compounds identified were arbutin (Ab), phenolic acids, and flavonoids - glycosides of flavones, and flavanones (Tables 1, 2; (Figure 1). However, Ab was identified only in sweet marjoram extract, where it reached 2%, and in common thyme extract (about 0.5%). Caffeic and rosmarinic acids were present in all of the studied extracts but lithospermic acid (LA) at higher amounts only in the wild thyme (over 6%) and peppermint extracts (2.5%). Much lower peaks were observed for other phenolic acids such as lithospermic acid B (syn. salvianolic acid B). The major flavonoid of ExTv, ExTs, and ExOm was luteolin-7-*O*-β-glucuronide (Lgr), while in peppermint it was eriocitrin (Er), followed by luteolin-7-*O*-rutinoside (Lr) and luteolin-7-*O*-β-glucuronide (Lgr). Additionally, apigenin-7-*O*-β-glucuronide (Agr) was identified and quantified in all aqueous extracts. The content of this flavone was relatively higher in sweet marjoram (1.5%) in comparison with the remaining extracts. Moreover, vicenin-2, a di-*C*-glucoside of apigenin (Engel et al., 2016) with interesting biological properties, was also detected in ExOm (1.2%). On the other hand, peppermint was the only extract containing other rutinosides (except for Er and Lr): hesperidin, diosmin, narirutin, and isorhoifolin, nevertheless their quantity did not exceed 0.1%. The other flavonoid glucosides, glucuronides, and rutinosides were detected only in very small or trace amounts. Generally, ExMp demonstrated the highest (poly)phenol content (PPs; ∼47%), followed by ExTs (∼37%) and ExTv (∼24%) with the lowest PPs detected in ExOm (∼18%). In ExMp, the flavonoid content (Fs; ∼39%) predominated over the phenolic acid level (PAs, ∼8%). Conversely, in ExTs and ExTv, phenolic acids dominated over flavonoids (∼26% and ∼16% vs. ∼11% and 8%, respectively). The lowest fraction of flavonoids was observed in ExOm (Table 2). The percentage of polyphenols in the extracts is consistent with previous data (Berdowska et al., 2013). The representative 2D HPLC-PDA chromatograms with the major components highlighted are illustrated in Figure 2.

**TABLE 1:** UHPLC-MS identification of Lamiaceae polyphenols.

No.	t_R_ (min)	[M-H]^−^ (*m/z*)	MS/MS (*m/z*)	Polyphenol	References	Extract (Ex)
*Hydroquinone derivative*						
1	3.63	271.0813	108/109	Arbutin	S	Om, Tv
*Phenolic acids – caffetannins*						
2	9.25	179.0358	135	Caffeic acid	S	Om, Tv, Ts, Mp
3	11.31	537.1042	493, 295, and 185	Lithospermic acid (Lithospermic acid A)	S	Ts, Mp
4	13.30	717.1455	519, 339, 321, and 295	Lithospermic acid B (salvianolic acid B)	Bodalska et al. (2020); Bodalska et al. (2021); Engel et al. (2016)	Om, Tv, Ts, Mp
5	12.88	359.0775	197, 179, 161, and 135	Rosmarinic acid	S	Om, Tv, Ts, Mp
*Flavones and flavanones*						
6	12.18	577.1558	269	Apigenin-7-O-rutinoside (isorhoifolin)	S	Mp
7	12.55	431.0996	269	Apigenin-7-*O*-β-glucoside	S	Om, Tv, Ts
8	12.68	445.0772	269	Apigenin-7-*O*-β-glucuronide	Bodalska et al. (2020); Bodalska et al. (2021); Engel et al. (2016)	Om, Tv, Ts, Mp
9	10.17	593.1507	503, 473, 383, and 535	Apigenin-6,8-di-*C*-glucoside (vicenin-2)	Engel et al. (2016)	Om
10	12.46	607.1668	299	Diosmetin-7-*O*-rutinoside (diosmin)	S	Mp
11	11.11	595.1672	287	Eriodictyol-7-*O*-rutinoside (eriocitrin)	S	Om, Tv, Ts, Mp
12	11.42	449.1088	287	Eriodictyol-7-*O*-β-glucoside	S	Tv, Ts, Mp
13	11.73	463.0862	287	Eriodictyol-7-*O*-β-glucuronide	Bodalska et al. (2020); Bodalska et al. (2021)	Om, Tv, Ts, Mp
14	12.41	609.1819	301	Hesperetin-7-*O*-rutinoside (hesperidin)	S	Mp
15	11.43	593.1507	285	Luteolin-7-*O*-rutinoside (scolymoside)	S	Om, Tv, Ts, Mp
16	11.71	447.0941	285	Luteolin-7-*O*-β-glucoside	S	Om, Tv, Ts, Mp
17	11.77	461.0737	285	Luteolin-7-*O*-β-glucuronide	S	Om, Tv, Ts, Mp
18	11.97	579.1709	271	Naringenin-7-*O*-rutinoside (narirutin)	S	Mp

S, authentic standard; Om, Origanum majorana L.; Tv, thymus vulgaris L.; Ts, thymus serpyllum L.; Mp, mentha × piperita L.

**TABLE 2:** Polyphenol contents (%) in dried aqueous extracts from sweet marjoram (ExOm), common thyme (ExTv), wild thyme (ExTs), and peppermint (ExMp) (as heatmap).

Compound	Symbol	HPLC-PDA		ExOm	ExTv	ExTs	ExMp
		λ (nm)	t_R_ (min)	% ± SD in dry aqueous extract (m/m)			
Arbutin	Ab	280	3.7	2.1 ± 0.03	0.5 ± 0.01	0	0
Caffeic acid	CA	320	8.3	1.1 ± 0.04	0.4 ± 0.02	0.3 ± 0.01	0.1 ± 0
Rosmarinic acid	RA	320	16.8	7.9 ± 0.31	15.1 ± 0.57	19.3 ± 0.8	5.9 ± 0.36
Lithospermic acid	LA	320	16.5	0	0	6.3 ± 0.12	2.5 ± 0.06
Eriodictyol-7-*O*-rutinoside	Er	280	12.1	0.3 ± 0.01	0.6 ± 0.01	0.3 ± 0.01	26.9 ± 0.6
Luteolin-7-*O*-rutinoside	Lr	360	12.4	< LQ	0.3 ± 0.02	0.2 ± 0.01	8.9 ± 0.31
Luteolin-7-*O*-β-glucuronide	Lgr	360	13.2	4 ± 0.13	6.6 ± 0.24	10.1 ± 0.27	2.8 ± 0.14
Apigenin-7-*O*-β-glucuronide	Agr^a^	360	15.8	1.5 ± 0.04	0.5 ± 0.01	0.4 ± 0.01	0.1 ± 0
Vicenin-2	V2^a^	360	6.9	1.2 ± 0.07	0	0	0
*Sum of phenolic acids*	PAs	Calculated		9 ± 0.35	15.5 ± 0.49	25.9 ± 0.51	8.4 ± 0.28
*Sum of flavonoids*	Fs			6.9 ± 0.26	7.9 ± 0.33	11 ± 0.27	38.7 ± 0.64
*Sum of polyphenols*	PPs			18 ± 0.53	24 ± 0.51	36.9 ± 0.42	47.1 ± 0. 7

a Calculated as Lgr.

LQ, limit of quantification; Ex, an extract of; Om, Origanum majorana; Tv, Thymus vulgaris; Ts, Thymus serpyllum; Mp, Mentha × piperita; n = 6.

### 3.2 Jurkat Apoptosis

Performed in this study FACS analysis with the application of a human Annexin V-FITC kit allowed for the discrimination between different Jurkat cells subpopulations: live (A-/PI-), early apoptotic (A+/PI-), late apoptotic/secondary necrotic (A+/PI+), necrotic or mechanically damaged (A-/PI+). Since in this study the influence of the examined substances on the degree of Jurkat T cells apoptosis was evaluated, two subpopulations of the cells were extracted and analyzed; percent of early apoptotic cells (A+/PI-), and percent of total apoptotic cells (the sum of early apoptotic (A+/PI-) and late apoptotic/secondary necrotic cells (A+/PI+)). However, since the total apoptosis levels reflected the early apoptotic changes, for graphical presentation total apoptosis was applied, termed just “apoptosis.”

#### 3.2.1 Modulation of Jurkat Apoptosis by Tv, Ts, Om, and Mp Extracts

**FIGURE 3:**
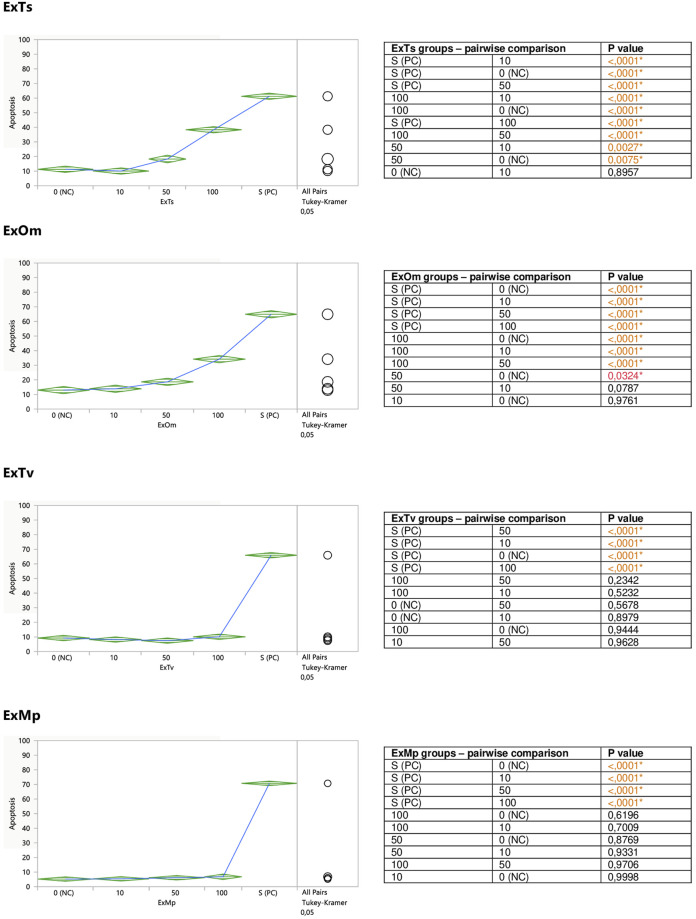
Direct effect of ExTv, ExTs, ExOm, and ExMp on apoptosis of Jurkat cells. 0 (NC) = negative control (untreated Jurkat cells); S (PC) = positive control (Jurkat cells treated with staurosporine); 10, 50, 100 = Jurkat cells incubated with a given extract at the concentration of 10, 50, or 100 μg/ml. Apoptosis = percent of apoptotic cells. Means diamonds illustrate ANOVA analysis (upper and bottom points are at 95% confidence points of each group). The circles on the right express significant differences between group means (if they do not overlap), or the lack of significant differences (if they overlap).

When the impact of dried aqueous extracts from *Thymus vulgaris* L. (ExTv), *Thymus serpyllum* L. (ExTs), *Origanum majorana* L. (ExOm), and *Mentha × piperita* L. (ExMp) on Jurkat cells was examined, a stimulatory effect on the cells’ apoptosis was observed in the case of two extracts; ExTs and ExOm. (Figure 3). The incubation of Jurkat cells with 50 and 100 μg/ml concentrations of ExTs significantly raised the percentage of the cells undergoing apoptosis, in comparison both with the control cells and the cells exposed to the lowest concentration of ExTs (10 μg/ml). This effect was the most evident at the highest concentration (2.9-fold increase in early apoptosis in comparison with the negative control (NC)), and even more pronounced when total apoptosis (early and late) was taken into account (3.4-fold increase in comparison with NC). Similarly, the exposition of Jurkat cells to both 50 and 100 μg/ml concentrations of ExOm resulted in a statistically significant increase in the number of apoptotic cells in the case of both early and total apoptosis in comparison with NC, demonstrating a 2.6-fold increase in total apoptosis at the greatest concentration.

The incubation of Jurkat cells with the remaining two extracts (ExTv and ExMp) exhibited no clear effect on the cells’ apoptosis (Figure 3). Slight fluctuations around the NC values were observed in the case of ExTv; the lower concentrations (10 and 50 μg/ml) tended to diminish the number of apoptotic cells, and the greatest concentration of 100 μg/ml increased the percent of apoptosis, but these effects were not statistically significant. ExMp demonstrated a mild tendency to increase the number of apoptotic cells at the two greatest concentrations.

**FIGURE 4:**
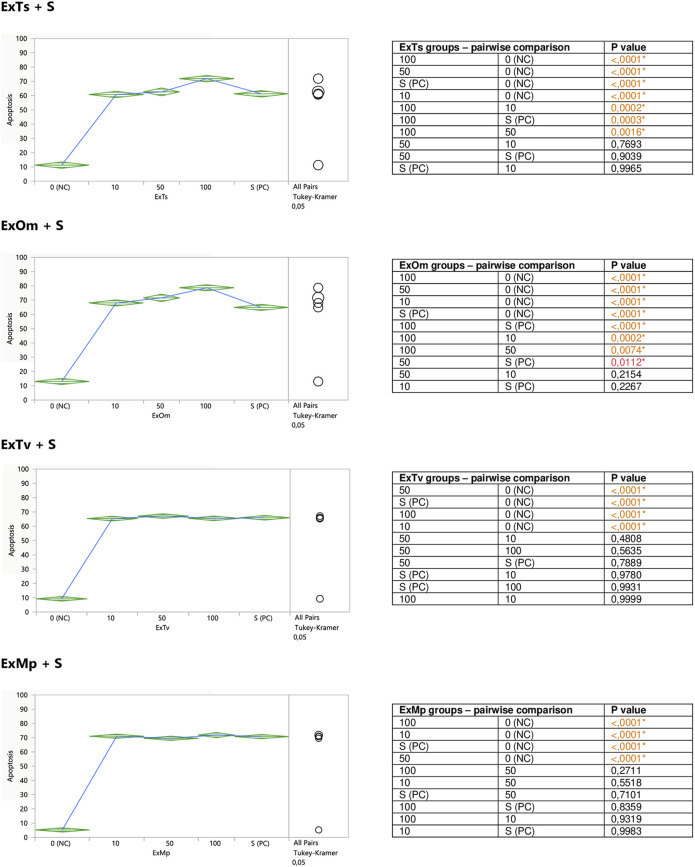
Modulatory effect of ExTv, ExTs, ExOm, and ExMp on staurosporine-induced apoptosis of Jurkat cells. 0 (NC) = negative control (untreated Jurkat cells); S (PC) = positive control (Jurkat cells treated with staurosporine); 10, 50, 100 = Jurkat cells incubated with a given extract at the concentration of 10, 50, or 100 μg/ml, and subsequently treated with staurosporine. Apoptosis = percent of apoptotic cells. Means diamonds illustrate ANOVA analysis (upper and bottom points are at 95% confidence points of each group). The circles on the right express significant differences between group means (if they do not overlap), or the lack of significant differences (if they overlap).

The aim of the second part of the experiment was to assess a putative modifying impact of the examined substances on the degree of staurosporine-induced apoptosis in Jurkat cells. For this reason, the cells were exposed to different concentrations of the extracts or polyphenolic compounds prior to the stimulation with staurosporine. Both ExTs and ExOm enhanced the proapoptotic effect of staurosporine at their highest concentrations of 100 μg/ml (Figure 4). Preincubation with ExTs increased the number of apoptotic cells by over 17% and the preincubation with ExOm demonstrated over 21% greater apoptosis degree, in comparison with the staurosporine-induced control (PC). However, this influence was observed only when total apoptosis was considered. Neither ExTv nor ExMp showed a modulatory effect on staurosporine-induced apoptosis (Figure 4).

#### 3.2.2 Modulation of Jurkat Apoptosis by CA, RA, LA, Lgr, Lr, Er, and Ab

**FIGURE 5:**
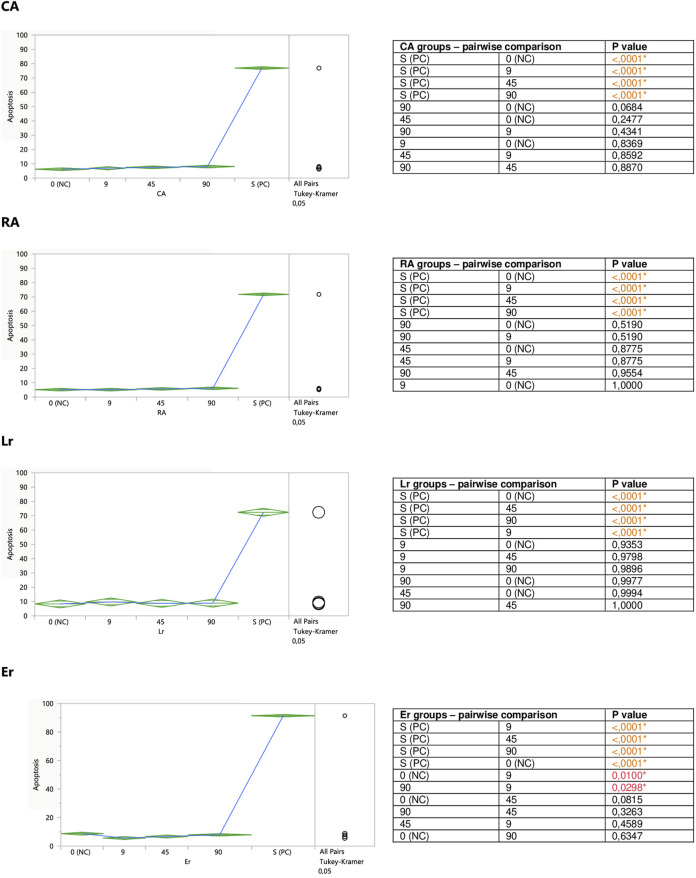
Direct effect of CA, RA, Lr, and Er on apoptosis of Jurkat cells. 0 (NC) = negative control (untreated Jurkat cells); S (PC) = positive control (Jurkat cells treated with staurosporine); 9, 45, 90 = Jurkat cells incubated with a given (poly)phenol at the concentration of 9, 45, or 90 µM. Apoptosis = percent of apoptotic cells. Means diamonds illustrate ANOVA analysis (upper and bottom points are at 95% confidence points of each group). The circles on the right express significant differences between group means (if they do not overlap), or the lack of significant differences (if they overlap).

Caffeic (CA) and rosmarinic (RA) acids, as well as luteolin-7-*O*-rutinoside (Lr) tended to induce apoptosis on their own (Figure 5), or enhance the staurosporine-induced apoptotic effect (Figure 7). However, the only statistically significant pro-apoptotic effect was observed when Jurkat cells were subjected to staurosporine action after the preincubation with CA and RA. Especially the first compound significantly raised the number of apoptotic cells by around 6 and 11% at 45 and 90 micromolar concentrations, respectively (when compared with PC). The impact of RA proved to be statistically significant only at the highest concentration, at which RA increased the number of apoptotic cells by about 7%.

**FIGURE 6:**
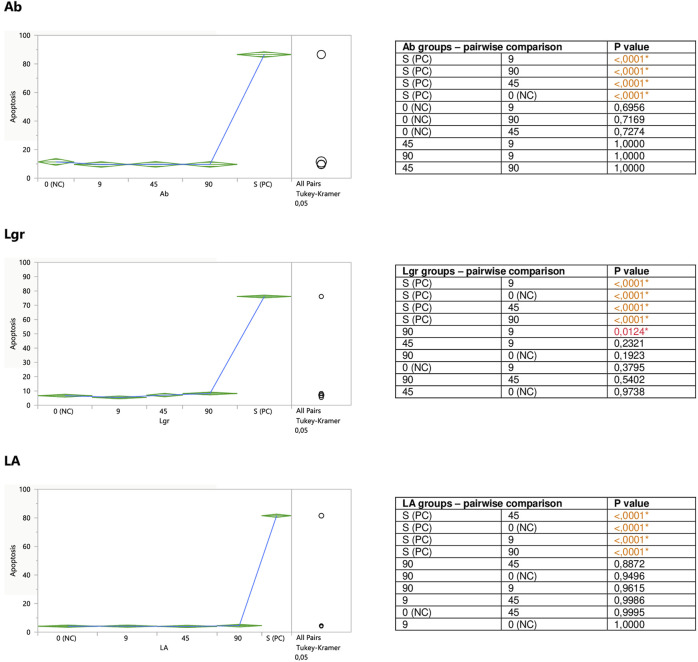
Direct effect of Ab, Lgr, and LA on apoptosis of Jurkat cells. 0 (NC) = negative control (untreated Jurkat cells); S (PC) = positive control (Jurkat cells treated with staurosporine); 9, 45, 90 = Jurkat cells incubated with a given (poly)phenol at the concentration of 9, 45, or 90 µM. Apoptosis = percent of apoptotic cells. Means diamonds illustrate ANOVA analysis (upper and bottom points are at 95% confidence points of each group). The circles on the right express significant differences between group means (if they do not overlap), or the lack of significant differences (if they overlap).

The incubation of unstimulated staurosporine Jurkat cells with eriodictyol-7-*O*-rutinoside (Er) resulted in a decrease in apoptosis level within the whole concentration range, although only the impact of the lowest 9 micromolar concentration was statistically significant and much stronger than 90 μM; inhibiting the apoptosis by around 35% (Figure 5). On the other hand, luteolin-7-*O*-glucuronide (Lgr), arbutin (Ab), and lithospermic acid (LA) did not modulate the apoptosis intensity (Figure 6).

**FIGURE 7:**
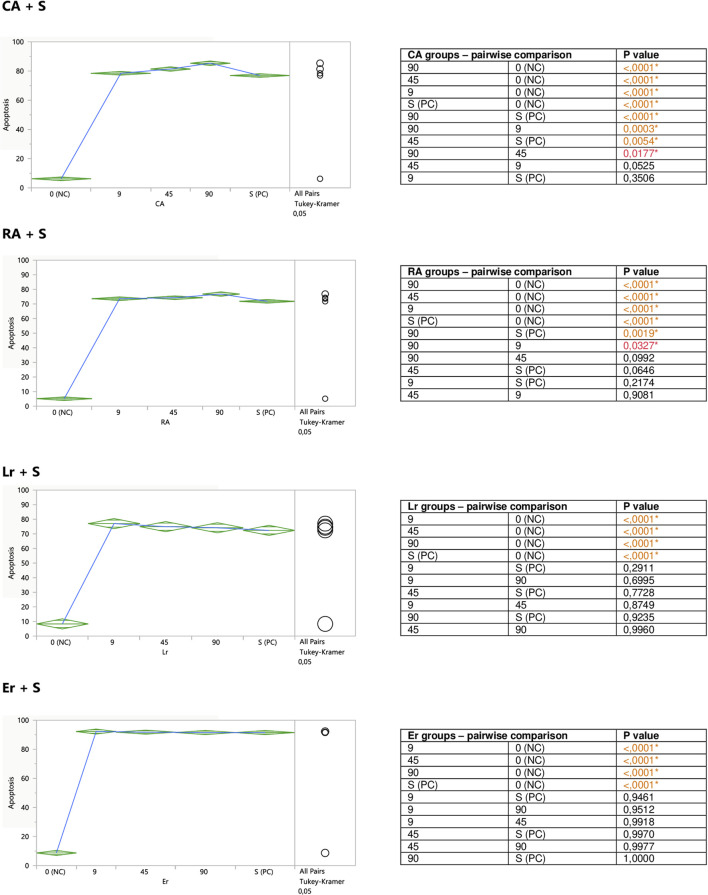
Modulatory effect of CA, RA, Lr, and Er on staurosporine-induced apoptosis of Jurkat cells. 0 (NC) = negative control (untreated Jurkat cells); S (PC) = positive control (Jurkat cells treated with staurosporine); 9, 45, 90 = Jurkat cells incubated with a given (poly)phenol at the concentration of 9, 45, or 90 μM, and subsequently treated with staurosporine. Apoptosis = percent of apoptotic cells. Means diamonds illustrate ANOVA analysis (upper and bottom points are at 95% confidence points of each group). The circles on the right express significant differences between group means (if they do not overlap) or the lack of significant differences (if they overlap).

**FIGURE 8:**
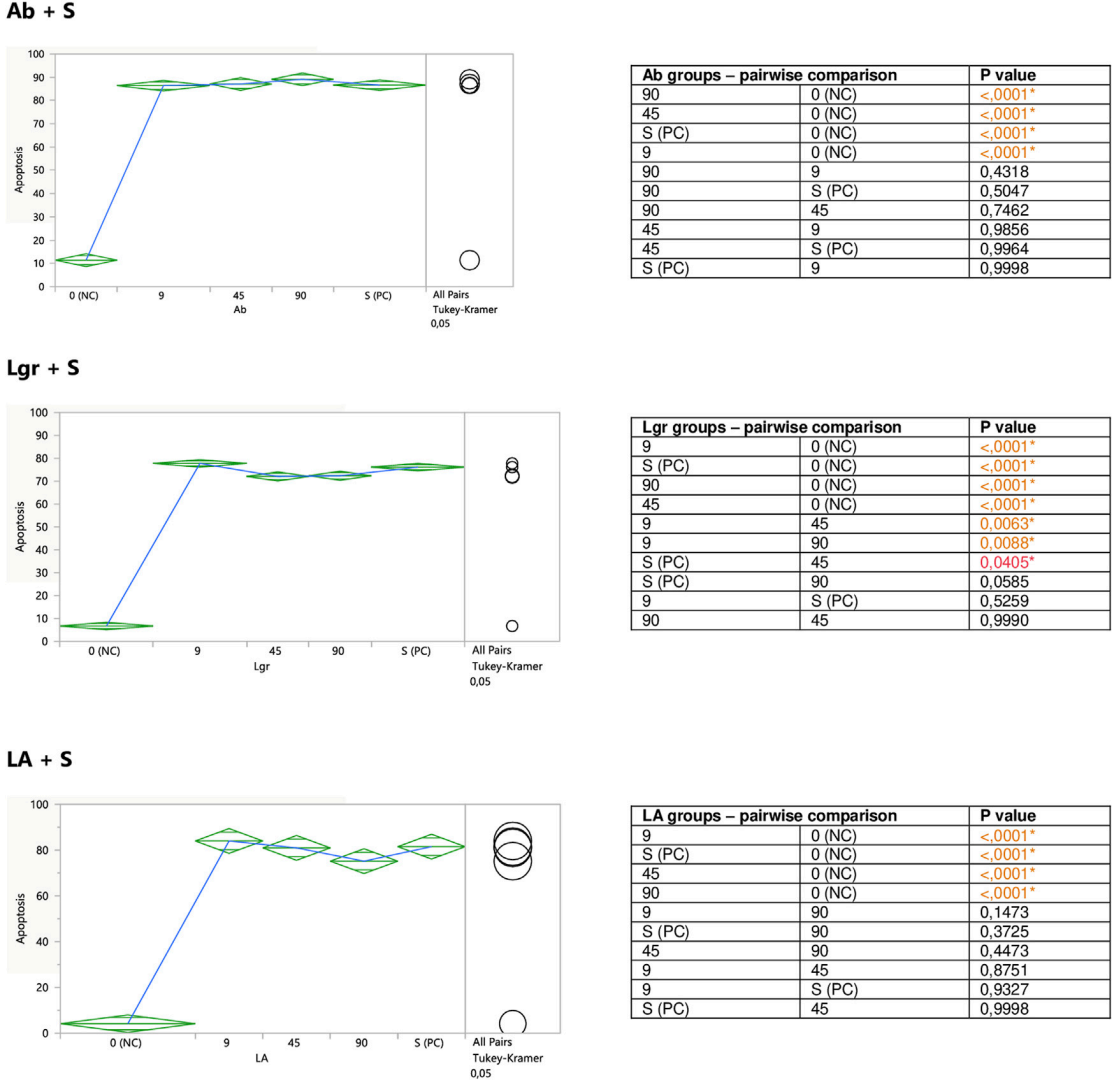
Modulatory effect of Ab, Lgr, and LA on staurosporine-induced apoptosis of Jurkat cells. 0 (NC) = negative control (untreated Jurkat cells); S (PC) = positive control (Jurkat cells treated with staurosporine); 9, 45, and 90 = Jurkat cells incubated with a given (poly)phenol at the concentration of 9, 45, or 90 μM, and subsequently treated with staurosporine. Apoptosis = per cent of apoptotic cells. Means diamonds illustrate ANOVA analysis (upper and bottom points are at 95% confidence points of each group). The circles on the right express significant differences between group means (if they do not overlap), or the lack of significant differences (if they overlap).

Er, Ab, and LA did not alter staurosporine-induced apoptosis in Jurkat cells, whereas Lgr seemed to weaken the pro-apoptotic action of staurosporine, diminishing the number of apoptotic cells by around 5% at higher concentrations (45 and 90 μM), but these effects maintained at the border of statistical significance (Figures 7, 8).

## 4 Discussion

From the observed here effects on Jurkat cells, the most pronounced was pro-apoptotic action of polyphenolic mixtures extracted from *Thymus serpyllum* L. and *Origanum majorana* L., as well as enhancement of staurosporine-induced apoptosis observed for caffeic acid and to the lesser extent for rosmarinic acid. In our previous work, the same extracts and polyphenols had been tested on human breast cancer cell lines resistant and non-resistant to Adriamycin (MCF-7/wt and MCF-7/Adr, respectively) with an MTT assay (Berdowska et al., 2013). Agreeably, the highest cytotoxicity against MCF-7/Adr had been observed in the case of ExOm and ExTs. However, in that previous study, ExTv had demonstrated comparable cytotoxicity in comparison with ExTs, whereas in the present experiment ExTv, in contrast to ExTs, did not show the proapoptotic effect on the Jurkat cell line, neither modulatory impact on staurosporine treated cells. The lack of effect on Jurkat cells treated or non-treated with staurosporine was also observed in the case of ExMp. This finding is closer to our previous observation when ExMp had exerted an only mild cytotoxic impact on MCF-7/Adr cells, in comparison with much higher ExOm toxicity. However, all four extracts in the previous study (at concentrations of up to 250 μg/ml) had increased the viability of MCF-7/wt cells (the regular breast cancer cell line, non-resistant to antibiotics). Therefore, no definite conclusions can be drawn on the influence of the tested extracts on these two cancer types. This is due to the fact that different cell lines and experimental procedures have been applied in this and the former studies. As the analysis of (poly)phenolic metabolites of the studied extracts demonstrated, illustrated here in Figure 1; Table 2, ExMp is the most abundant in (poly)phenolic compounds (PPs reaching 47%), whereas ExOm contains only around 18% PPs. Additionally, ExMp is the richest in flavonoid glycosides (Er, Lr, and Lgr) when compared with the remaining three extracts, and shows an especially high concentration of Er reaching 27%, whereas in the other extracts it does not exceed 1%. Reversely, ExMp contains the least caffeic acid and its esters (CA + RA + LA), the subfraction most abundant in ExTs containing the greatest amounts of RA and LA. Since both CA and RA demonstrated the strongest pro-apoptotic activity in the present study, it might be hypothesized that this is CA-RA (∼ 20%) subfraction that mostly contributes to the ExTs pro-apoptotic effect. However, if this was the case, we would also have expected a pro-apoptotic action exerted by ExTv, whereas comparable to ExTs pattern was actually observed for ExOm containing lower phenolic acids percentage (like in ExMp PAs subfraction). The lack of ExMp effect on Jurkat cells apoptosis may be associated with the somewhat opposite actions of Er and Lr; whereas pure Er demonstrated anti-apoptotic action, pure Lr tended to stimulate apoptosis of Jurkat cells. Therefore, when present in the ExMp mixture they might have mutually abolished their actions giving a resultant in the shape of no effect at all. On the other hand, when Lr and Er had been tested on breast cancer cell lines, Er demonstrated a greater level of toxicity against both MCF-7/wt and MCF-7/Adr than Lr. The most toxic against both MCF-7 cell lines had been RA and LA, whereas in the present study the strongest pro-apoptotic impact was observed in the case of CA, followed by RA, whereas LA did not affect Jurkat cells apoptosis. Ab being the least cytotoxic against breast cancer cells, exhibited no impact on Jurkat cells, similarly to LA.

The comparison of biological activities exerted by plant extracts (containing mixtures of compounds) with actions of isolated substances is a problematic issue. On one hand, there is a strong tendency aimed at optimization of techniques to isolate/produce the desired compound of the highest possible degree of purity; precisely described structurally and functionally, which after further testing gives rise to the generation of a medicinal drug. On the other hand, it seems that in many cases the purification process leads to the weakening of desired biological effects. Therefore, the strategy associated with the assessment of metabolites properties in mixtures should be regarded as an alternative approach in the search for the natural substances which can be applied in many areas including adjuvant therapies. Such an approach has been lately presented in the experimental work of Ouedrhiri et al. (2021), who aimed at the optimization of the mixture design technology to achieve the best antioxidative effect of essential oil fractions from chosen Lamiaceae herbs. In both studies on human breast cancer and human leukemia cells, the most beneficial effects have been observed for polyphenolic mixtures; the greater cytotoxic impact of the studied extracts on the cells with developed multidrug resistance in the first case (Berdowska et al., 2013), and the most prominent anti-apoptotic impact of ExTs and ExOm, when compared with the effect of pure polyphenols. Nevertheless, as discussed above, likewise in the previous study (Berdowska et al., 2013), the impact of extracts did not directly reflect their (poly)phenolic composition, which might be explained by different types of interactions among the constituents in the mixtures (additive, synergistic, contradictory, enforcing, or attenuating one another activities, etc.) but also by the interference of unidentified earlier compounds. For example, the pro-apoptotic effect of ExOm may be partially ascribed (except for RA-CA subfraction), to vicenin-2 (not detected in the remaining extracts) whose anti-cancer properties have been demonstrated in some studies (Nagaprashantha et al., 2011; Li et al., 2021).

Stronger biological effects of mixtures of compounds have been also observed in some other studies (Morré et al., 2003; Seeram et al., 2005; Adams et al., 2006). Reversely, since some of the tested here compounds such as flavonoid glycosides (Er, Lr, Lgr) belong to complex molecules, they may undergo hydrolysis in the experimental conditions of cell cultures. Therefore, the observed on the cell lines effects might be associated partially with their released products. Such a phenomenon has been recognized in the experiments on ellagitannin (punicalagin) by Larrosa et al. (2006).

Among the studied phenolic compounds, the most thoroughly investigated, in regard to their health-beneficial properties, are probably caffeic and rosmarinic acids, recently addressed in some review studies. (Habtemariam et al., 2017; Colica et al., 2018; Ngo et al., 2018; Swamy et al., 2018; Elufioye and Habtemariam, 2019; Espíndola et al., 2019; Ghasemzadeh Rahbardar et al., 2020; Luo et al., 2020; Silva et al., 2020; de Araújo et al., 2021; Ekeuku et al., 2021; Hitl et al., 2021; Khan et al., 2021; Muhammad Abdul Kadar et al., 2021). For example, CA and its more studied derivative caffeic acid phenethyl ester demonstrate anti-atherosclerotic as well as blood pressure decreasing properties, thus showing potential for cardiovascular disease treatment (Silva and Lopes, 2020). CA putative protection against metabolic syndrome and associated disorders such as insulin resistance and diabetes has been discussed by Muhammad Abdul Kadar (2021). Additionally, CA due to its bone remodeling activities is a promising agent in bone diseases therapies (Ekeuku et al., 2021). An application of CA and its derivatives in infectious disorders has been discussed by Khan et al. (2021), and CA anti-cancer activities (with respect to hepatocarcinoma) have been considered by Espíndola et al. (2019). Anti-inflammatory properties of RA have been addressed by Luo et al. (2020), where its capacity to attenuate arthritis, colitis, dermatitis and similar conditions has been discussed. The hepatoprotective properties of RA have been addressed by Elufioye and Habtemariam (2019). Moreover, like in the case of CA, also RA is a promising compound in protection against diabetes (Ngo et al., 2018), as well as neoplasia developments (Swamy et al., 2018). Among the mentioned above CA and RA health-beneficial properties, their well-recognized functions are anti-cancer activities. They have been demonstrated to inhibit neoplastic cell development via different mechanisms, including the stimulation of cell death through pro-apoptotic actions. Pro-apoptotic effects of CA and RA on Jurkat cells were also observed in the present study, where both compounds enhanced staurosporine-induced apoptosis.

The limitation of this study is the narrow concentration range of the evaluated extracts/compounds which was conditioned by technical constraints of the experimental protocol. A sufficient number of Jurkat cells in each sample had to be achieved, as well as repetitions of experiments to reach statistical significance were necessary. Two experimental patterns conducted in parallel (with and without staurosporine preincubation) doubled the number of occupied wells in each 24-well plate. All of these characteristics technically restricted the number of the available wells; therefore to perform each experiment in triplicate, only three different concentration levels of the studied substances were tested

The strength of this study is the novelty in the evaluation of the impact of specifically characterized dried aqueous extracts from Mp, Ts, Tv, and Om on Jurkat cells with respect to their apoptosis modulatory properties, as well as the comparison of their effects with their (poly)phenolic components. The presented here experimental model is unique with respect to the comparison of the actions of single molecules with their properties in mixtures, as well as the applied cell line. Concluding from the literature search, there have been only a few experiments investigating the effects studied in this work substances on Jurkat cells apoptosis. On the other hand, a variety of experimental designs makes it difficult to compare the obtained here results with the data from other teams. For example, when it comes to Mentha genus species, their anti-cancer properties have been demonstrated both in *in vitro* studies and clinical trials (Anwar et al., 2019). However, various mint species (*Mentha piperita, Mentha spicata, Mentha arvensis, Mentha longifolia*), different cell lines (Hela—human malignant cervix carcinoma, Hep2—human laryngeal carcinoma, Vero—green African monkey kidney), disparate formulations in extract preparations (e.g., alcoholic vs. aqueous), and different tests and protocols applied for the estimation of cytotoxic/antiproliferative potential, causes drawing unequivocal conclusions really problematic. Whereas in the present study, no apoptosis-modulatory impact was observed when ExMp was tested on Jurkat cells, in the study by Jain et al. (2011), peppermint extracts demonstrated proapoptotic activity in the same cell line and with the application of the same test. Nevertheless, much stronger effects were obtained by the authors when chloroform or ethyl acetate was used for extract preparations, in comparison with water extract, but what is more, the authors evaluated extracts within different concentration ranges. In the present study 0.01–0.1 mg/ml concentrations were tested, whereas in Jain’s experiments the concentration range was much higher (1–100 mg/ml). Therefore, it might be suggested that, despite the observed here lack of effect for low concentrations of peppermint aqueous extracts, if a different extraction procedure and greater concentrations had been applied, then a pro-apoptotic effect might have been observed. The common thyme extract effects on Jurkat cells have been assessed in another study (Esmaeilbeig et al., 2015), in which the authors found the cytotoxic impact of Tv on the cells, which seems to stand in opposition to the present experimental finding. However, the authors did not evaluate the level of apoptosis but the viability of the cells with the MTT assay. Additionally, they incubated the cells with Tv extract for 48 h (whereas in this study the incubation time was up to 20 h), and they prepared methanol, not aqueous Tv extract. Therefore, this putative discrepancy mostly derives from the differences in the experimental protocol. Sweet marjoram ethanol extract has been tested on Jurkat cells by Abdel-Massih et al. (2010), who observed cytotoxic and pro-apoptotic impact, similar to the present study. However, the authors applied longer incubation times (48 and 96 h), as well as greater concentrations. Nevertheless, the extracts derived from *Origanum majorana* L. seem to demonstrate the most promising anti-cancer effects in comparison with the remaining three Lamiaceae species, which had been also observed in our previous experiments on human breast cancer cells (Berdowska et al., 2013).

When it comes to polyphenolic compounds studied in the present experiments, rosmarinic acid has previously demonstrated pro-apoptotic activity on Jurkat cells (Hur et al., 2004; Kolettas et al., 2006). Hur et al. (2004) observed that RA (at 30 µM concentration) induced apoptosis in 37.8–53.6% of Jurkat cells after 24 h incubation, and in 98.3% of the cells after 48 h incubation. The authors reported the mitochondrial pathway involvement in the mechanism of apoptosis induction because it was associated with mitochondrial membrane depolarization and the release of cytochrome C, and the participation of caspases 3 and 8. Accordingly, Kolettas et al. (2006) demonstrated both dose-dependent inhibition of Jurkat cells proliferation and induction of apoptosis by RA. The authors showed RA-stimulated downregulation of cyclin D3 and p21Cip1/Waf1 and upregulation of p27Kip1, which suggested the cell growth arrest at the G1/S phase. Additionally, they observed that the induction of apoptosis by RA correlated with the suppression of Bcl-2. Therefore, RA seems to be able to stimulate apoptosis of Jurkat cells *via* the reduction of anti-apoptotic Bcl-2 proteins, thus activating the mitochondrial pathway. In the present study, RA demonstrated a tendency to stimulate apoptosis in Jurkat cells, but this effect reached statistical significance only at 90 micromolar concentration in the cells treated with staurosporine, hence reinforcing the pro-apoptotic impact of staurosporine by increasing the number of apoptotic cells by 7%. The weaker pro-apoptotic effect of RA observed here when compared to Hur et al. (2004) may be ascribed to the shorter incubation time; in this study, it was 20 h, whereas in the Hur et al. (2004) experiments the incubation time was longer reaching 24 and 48 h. Since the authors observed a much greater effect after 48 h, it might be suggested that RA requires more than 20 h to exert its pro-apoptotic effect.

In conclusion, it might be hypothesized that these are rather (poly)phenolic extracts that demonstrate required properties against pathological cells than purified compounds. In the present experiment the most beneficial pro-apoptotic actions against human leukemia Jurkat cells have been observed in the case of dried aqueous extracts from *Thymus serpyllum* L. and *Origanum majorana* L. Less evident pro-apoptotic effects were demonstrated for purified (poly)phenolic compounds, namely caffeic and rosmarinic acids, which enhanced especially staurosporine-induced apoptosis. Nevertheless, due to the abovementioned limitation of the present study, if longer incubation time and greater concentrations of the tested compounds had been applied, stronger effects might have been observed. Therefore, the present experiments may be considered rather as a pilot study to encourage their continuation with the aim to gain more definite conclusions.

## Data Availability

The raw data supporting the conclusions of this article will be made available by the authors, without undue reservation.
